# Is statin-modified reduction in lipids the most important preventive therapy for cardiovascular disease? A pro/con debate

**DOI:** 10.1186/s12916-016-0550-5

**Published:** 2016-01-14

**Authors:** FD Richard Hobbs, Maciej Banach, Dimitri P. Mikhailidis, Aseem Malhotra, Simon Capewell

**Affiliations:** Nuffield Department of Primary Care Health Sciences, Oxford, UK; Department of Hypertension, Chair of Nephrology and Hypertension, Medical University of Lodz, Lodz, Poland; Department of Clinical Biochemistry, Royal Free Hospital Campus, University College London Medical School, University College London, London, UK; Frimley Park Hospital, Surrey, UK; Department of Public Health and Policy, University of Liverpool, Liverpool, UK

**Keywords:** Statins, Prevention, Cardiovascular disease, Debate, Evidence

## Abstract

The most prescribed medications in the world are statins, lipid modifiers that have been available for over 25 years and amongst the most investigated of all drug classes. With over a million patient years of trial data and publications in the most prestigious medical journals, it is remarkable that quite so much debate remains as to their place in healthcare. They have had a bittersweet passage, with vocal concerns over their possible risks, from suicide to cancer, and allegations that they do not work in women or the elderly, to statements that the whole published dataset, on over 200,000 patients consenting to enter trials, was fatally compromised by being industry-funded by and large. On the other side, there have been billions of dollars spent on generating their evidence base followed by promotion which has returned that investment many times over in profits, and a powerful scientific lobby that argue they are wonder drugs and that continued nihilism on their value risks patient lives. So who is right?

## Introduction

**Fig. 1:**
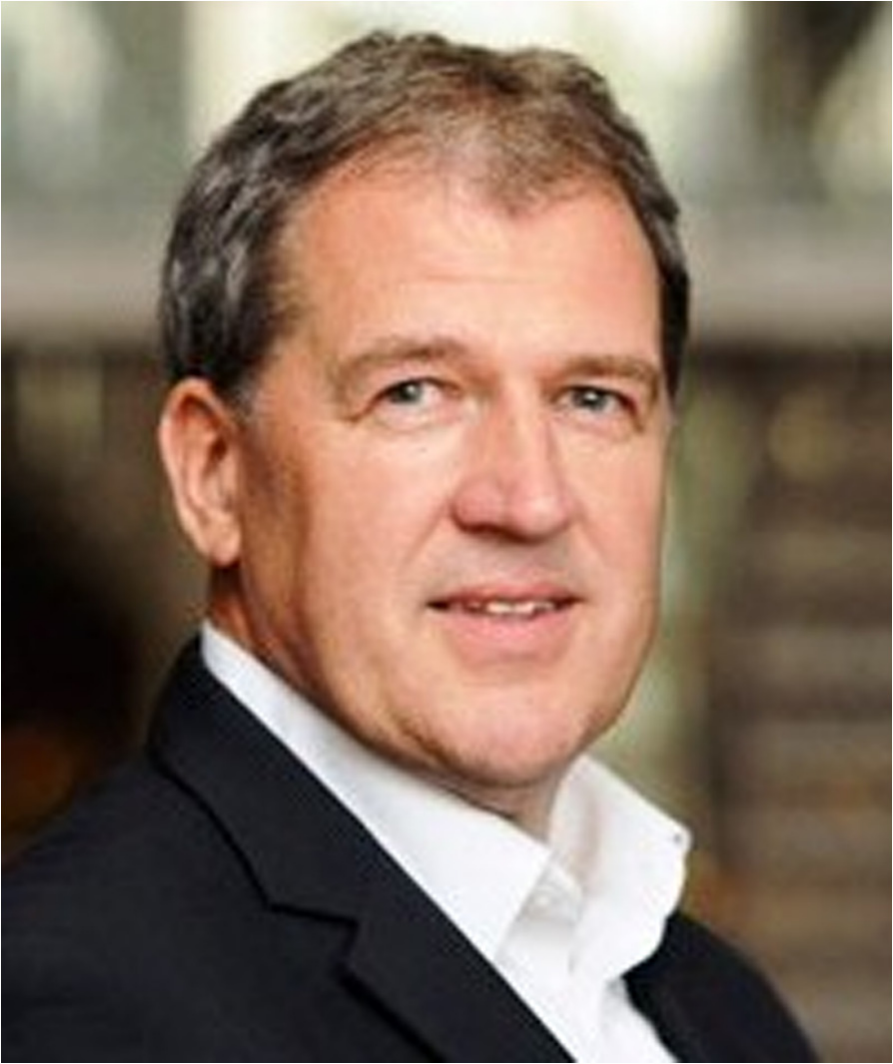
FD Richard Hobbs is Professor of Primary Care Health Sciences at the Nuffield Department of Primary Care Health Sciences, Oxford, UK. He is also Director of the National Institute for Health Research (NIHR) Collaboration for Leadership in Applied Health Research and Care, and the NIHR School for Primary Care Research. Professor Hobbs is a longstanding part-time practising general practitioner (GP) serving a deprived inner-city population. His research interests focus on cardiovascular epidemiology and clinical trials, particularly relating to vascular and stroke risk, and heart failure

FD Richard Hobbs (Fig. 1)

In this Forum article, Banach and Mikhailidis argue that statins are well tolerated and effective in primary and secondary prevention of all the main cardiovascular events. They quote data from many trials, especially the Cholesterol Treatment Trialists’ (CTT) Collaboration. They discuss the reported high rates of adverse events associated with statins, but argue that these can be ameliorated by certain strategies to help patients stay on medication. They also tackle the perceived risks of dropping low-density lipoprotein cholesterol (LDL-C) too low, using medication on top of statins, namely ezetimibe and proprotein convertase subtilisin/kexin type 9 (PCSK9) monoclonal antibodies; the latter showing no difference in adverse events with patients achieving on-treatment LDL above or below 25 mg/ml (0.65 mmol/l), a level that would have been unachievable a few years ago. Talking of the event and mortality benefits seen in primary prevention of cardiovascular disease (CVD), they nonetheless highlight that much residual risk remains for patients, i.e. despite their potency, about 75 % of events continue to occur. They close by citing basic scientific evidence of the anti-inflammatory effects of statins on endothelial plaque.

For Malhotra and Capewell, these same data elicit a very different response. Whilst acknowledging the unequivocal benefit of statins in secondary prevention of CVD, they dispute the benefits in primary prevention, stating that there is no mortality benefit and the reductions in CVD events are not accompanied by overall reductions in disease. They further undermine the case for statins by arguing that adverse events are much underreported, or even designed out, in trials, including the CTT meta-analyses. They cite data that statins increase rates of type 2 diabetes and further quote observational data which suggest that 60 % of the half of patients who stop statins after a year of use do so because of adverse effects. They go on to argue that dietary changes are much more important for primary prevention of CVD, with a huge evidence base.

So how does the bewildered clinician make recommendations to patients when specialists disagree on the evidence base so fundamentally? Of course evidence is rarely complete and increasingly it is important for us to try and ‘triangulate’ data from trials with real-world data, to test whether spectrum or selection bias was possibly influential in trial populations. The limitations of trial data (run-in phases, potentially select populations included, primary focus on effectiveness, etc.) are similarly a major issue for observational data (less reliable data, can only test associations, selective populations, etc.). Our authors agree that statins have high value in secondary prevention of CVD, but disagree on the relative importance of side effects in patients taking them.

There is no doubt that the greatest risk with statins is rhabdomyolysis, occurring in 1 in 10,000 patients [1], but this is 400 times less likely to happen in patients than a gastrointestinal (GI) bleed (and 500 times less likely fatal) in those taking low-dose aspirin, an over-the-counter medication. Furthermore, this important but very rare risk is mainly confined to the sick elderly, should be diagnosed quickly, and usually settles quickly on statin cessation. However, this important risk probably amplifies the increased muscle symptoms seen with statins in the general population; muscle symptoms are a very common complaint in life and the connection with statins is widely made in the media. Analysis of routine patient data in case-control analyses suggests that myopathy rates may be doubled by statin use [2]. But such observational data are not without confounding factors. Does this distort patient perception of statins and indeed adherence to them? Adjusting reported adverse event rates in trials for the rates observed in the placebo arms of 14 primary prevention trials (46,262 participants) showed no statin-attributable symptoms, including muscle effects, although asymptomatic liver transaminase elevation was 0.4 % more frequent with statins across all trials. Serious adverse events and withdrawals were similar in both arms [3]. The regulatory drug package inserts should present such placebo-adjusted adverse event rates, in the same way that they report treatment effects over and above any placebo benefits (observed in most trials).

The other ‘big’ safety issue is the increased rate of diabetes by an absolute risk of 0.5 % (95 % CI 0.1–1 %, *P* = 0.012) [3]. However, outcomes for those who progressed from an HbA1c below to above the diagnostic threshold to define new onset diabetes were the same as those not developing new onset diabetes in the trials (and we know that patients with established diabetes get even bigger risk reductions from statins in placebo-controlled trials). This is not a reason to be concerned over statins in ‘eligible for treatment’ populations.

We are unlikely to quantify adverse events much further – there is inevitable subjective weighting and interpretation of the data, which will always attract those that want to argue from one or other standpoint – and there is probably too much sediment to ever clear now.

As for benefits, this should be easier – as if! Personally, I think the data strongly point to a consistent relative risk reduction of statins across all lipid levels in contemporary populations; we have a very abnormal lipid phenotype in adults in most countries and it is therefore cost effectiveness (i.e. the absolute risk in populations) or disutility/utility arguments that should prevail. The National Institute for Health and Care Excellence (NICE), United States and European guidelines believe that the low cost of statins now means that those down to 10 %, 7.5 % or 15 % 10-year calculated CVD risk, respectively, are eligible to be offered a statin. Whether they take one is therefore a patient choice issue and we should not offer or attempt to cajole patients one way or another. For some patients, their fear of vascular disease will dominate and they may want a statin regardless of calculated risk. For others, the risk of symptoms will negate any perceived gain. What we should not pretend though is that personal CVD risk can be individually determined – our tools are crude and work at the population not personal level. Many people at low calculated risk will die prematurely; indeed, what happened to our parents may be a very significant guide and that is not incorporated into scores. Although it may be an accurate interpretation that mortality benefits were not seen for low-risk primary prevention in the trials, there were mortality gains across all primary prevention eligible patients, and the trials only look at 5 years’ follow-up. The available evidence suggests that a mortality gain would emerge for almost everyone using a statin above a certain risk level (such as simply over 55 years old), but our health systems might not want to pay for that benefit or patients might not want to comply with chronic medication long enough to gain.

Perhaps we should listen more to what really concerns our patients and then base treatment recommendations on their preferences, without unduly swaying them against or for a statin through our own perceptions of the huge evidence base of statins. There is never likely to be another area of healthcare with as much evidence as relates to statins and CVD, and yet clinicians cannot even agree here. We all have competing interests in this debate.

## Is statin-modified reduction in lipids the most important preventive therapy for cardiovascular disease? Yes!

**Fig. 2:**
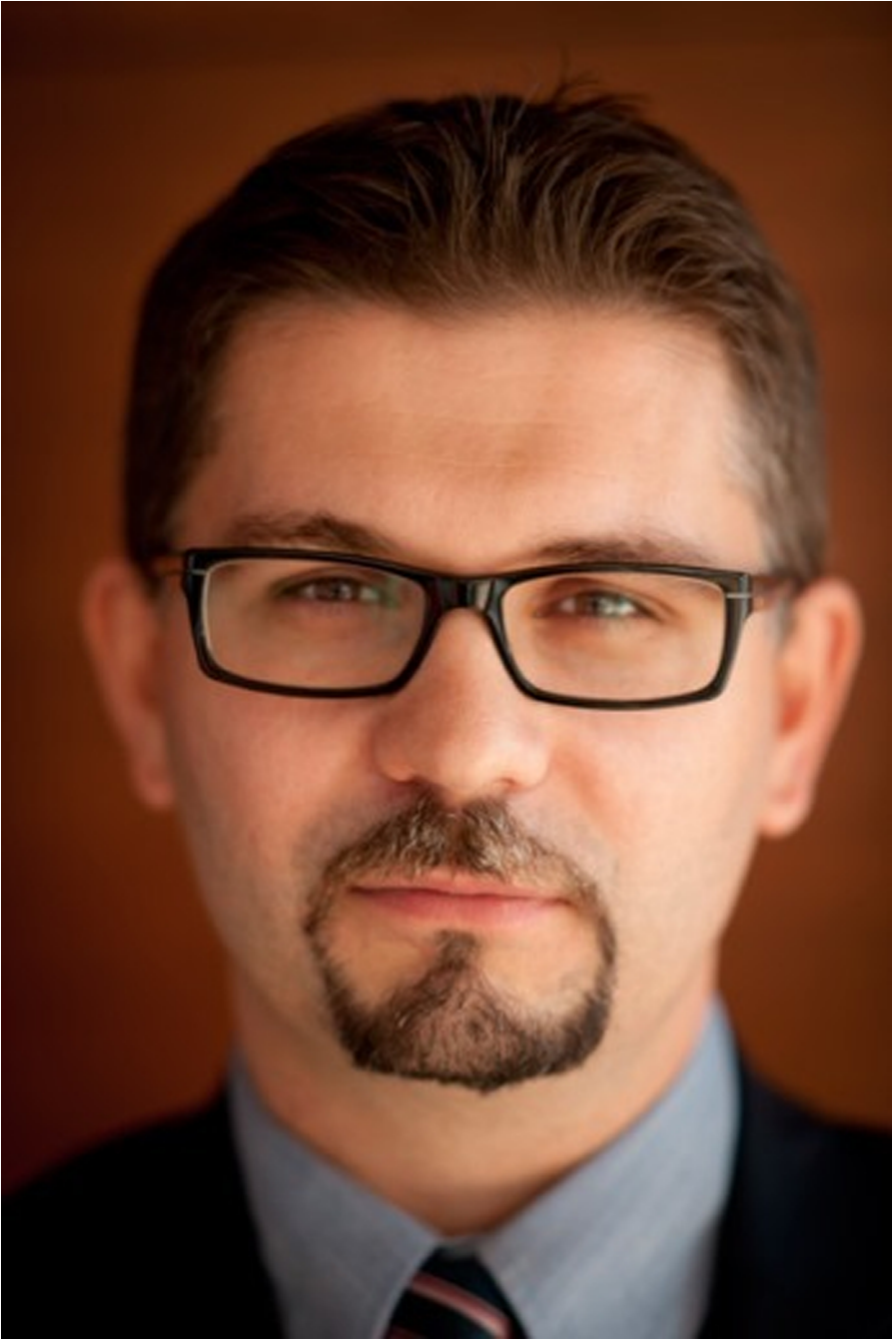
Maciej Banach is Professor of Cardiology at the Medical University of Lodz and President of the Polish Mother’s Memorial Hospital Research Institute, Lodz, Poland. He is also Visiting Professor at the University of Alabama at Birmingham, Birmingham, AL, USA, and Victor Babes University of Medicine and Pharmacy, Timisoara, Romania. He is a member of the Executive Committee of the European Atherosclerosis Society (EAS), Founder and Head of the Polish Lipid Association (PoLA), and the founder of the Lipid and Blood Pressure Meta-analysis Collaboration (LBPMC) Group. His main area of research concerns hypertension, lipid disorders, dyslipidemia therapy and new drugs in CVD therapy

**Fig. 3:**
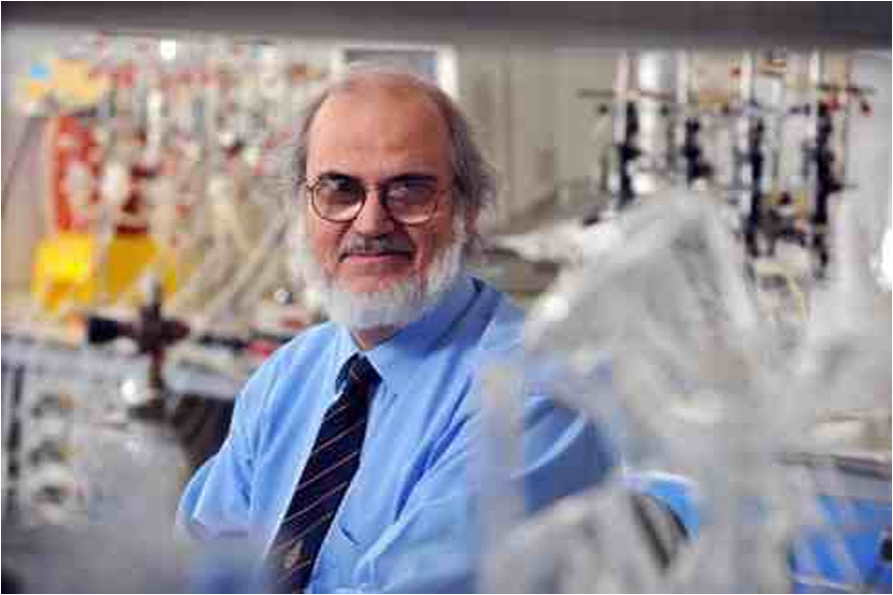
Dimitri P. Mikhailidis is Reader and Honorary Consultant at the Department of Clinical Biochemistry (Vascular Disease Prevention Clinics), Royal Free Hospital Campus, University College London Medical School, University College London, London, UK. He is also Visiting Professor at the Robert Gordon University, Aberdeen, Scotland. His main clinical interest is vascular disease prevention, and his research interests include lipids and atherothrombosis

Maciej Banach and Dimitri P. Mikhailidis (Figs. 2 and 3)

Statins are commonly prescribed drugs [4] that are well tolerated and effectively prevent cardiovascular events both in primary and secondary prevention [5, 6]. These drugs play an essential role in patients with CVD, as they significantly reduce the risk of acute myocardial infarction (AMI), cardiovascular revascularization, stroke, cardiovascular mortality and all-cause mortality [6, 7]. It has also been shown that these effects, as well as other benefits, might be observed irrespective of LDL-C goals achieved [6, 7]. In a study involving 1,054 patients with AMI who had baseline LDL-C levels <70 mg/dl (1.8 mmol/l), further statin therapy significantly reduced the risk of the composite primary endpoint (the composite of 1-year major adverse cardiac events, including death, recurrent myocardial infarction, target vessel revascularization and coronary artery bypass grafting) by 42 % (*P* = 0.015) [8]. Statin therapy also reduced the risk of cardiac death (by 53 %, *P* = 0.031) and coronary revascularization (by 55 %, *P* = 0.013).

In secondary prevention, ‘the lower the better’ phenomenon has been widely discussed since important studies such as the Pravastatin or Atorvastatin Evaluation and Infection Therapy-Thrombolysis in Myocardial Infarction 22 (PROVE-IT TIMI 22) [9] trial. The debate has been associated with some doubts concerning the additional cardiovascular efficacy as well as safety of further lowering of LDL-C to levels even below 40 and 30 mg/dl (1.0 and 0.8 mmol/l) [10]. These doubts have been clarified by the IMProved Reduction of Outcomes: Vytorin Efficacy International Trial (IMPROVE-IT) [11] and the results of studies assessing PCSK9 inhibitors [12]. The IMPROVE-IT trial included 18,144 patients who were hospitalized for acute coronary syndrome (ACS) and had LDL-C levels of 50–100 mg/dl (1.3–2.6 mmol/l) if they were receiving lipid-lowering therapy or 50–125 mg/dl (1.3–3.2 mmol/l) if they were not receiving lipid-lowering therapy. The combination of simvastatin (40 mg) and ezetimibe (10 mg) was compared with simvastatin (40 mg) and placebo. The median LDL-C level during the study was 53.7 mg/dl (1.4 mmol/l) in the simvastatin-ezetimibe group compared with 69.5 mg/dl (1.8 mmol/l) in the simvastatin monotherapy group (*P* <0.001) [11]. The Kaplan–Meier event rate for the primary endpoint at 7 years was 32.7 % in the simvastatin-ezetimibe group compared with 34.7 % in the simvastatin monotherapy group (absolute risk difference, hazard ratio (HR) 0.936, *P* = 0.016; number needed to treat (NNT) 50). Rates of therapy-related adverse effects (AEs) were similar in the two groups [11]. The IMPROVE-IT trial confirmed the efficacy and safety of intensive LDL-C-lowering in patients with ACS. Safety of the very low concentrations of LDL-C was also confirmed in the ODYSSEY LONG TERM trial with alirocumab added to statins, where no differences in AEs between the group with LDL-C below and over 25 mg/dl were noted [12, 13]. The available studies have also shown that the effectiveness of statin therapy, especially high-intensive regimens with rosuvastatin 20–40 mg and atorvastatin 40–80 mg, could be very high, and even in >90 % of patients we might achieve lipid goals [14]. Additionally, statin therapy might not only stabilize an atheroma plaque, but also reduces its volume as well as favourably influencing other parameters of its morphology. In the recent Lipid and Blood Pressure Meta-analysis Collaboration (LBPMC) Group meta-analysis [15] we showed that statin therapy significantly reduces plaque volume (standard mean difference (SMD) −0.137, *P* = 0.023), external elastic membrane volume (−0.097, *P* = 0.027), fibrous plaque volume (−0.129, *P* = 0.045) and increases dense calcium volume (0.229, *P* = 0.043).

However, despite this well-established role of statin therapy in secondary prevention patients, statin use is also associated with AEs and residual risk [16, 17]. Statin-related AEs might impair the effectiveness of statin therapy. Most of the AEs are muscle-related; however, there are also other statin-associated AEs, such as sleep disorders, hair loss, gastroenterological disorders or cognitive disorders, but for many of them causality has still not been confirmed [16, 18]. It is relevant to note that patients with statin-related side effects at baseline were excluded from the trials and that knowledge is mainly based on observational cohort studies, which probably overestimate the number of patients with AEs after statin therapy [16, 18]. Statin intolerance is a real phenomenon, which can affect up to 15 % of patients, and knowledge of the most common AEs associated with statin therapy is crucial to ensure effective treatment of lipid disorders by reducing the risk of discontinuing statin therapy and improving therapy adherence [16, 18]. However, with careful management (take your time approach) we might successfully treat over 90 % of the patients previously reporting statin intolerance [16, 18].

The issue of residual risk has been widely discussed for many years, each time with the occasion of new drug development for dyslipidaemic patients [19]. According to available studies residual risk involves 50–70 % of patients treated with statins, and recent data from the EUROASPIRE IV survey suggest that despite the fact that the majority (87 %) of secondary prevention patients now receive a statin, only 21 % of high-risk patients have LDL-C levels below 70 mg/dl (1.8 mmol/l) and 58 % below 100 mg/dl (2.5 mmol/l) [20]. Several factors can influence residual risk; this is associated with patients at very high risk with concomitant diseases and polypharmacy, and the problem of significant underuse of high doses of statins – in many European countries as well as in the United States atorvastatin 80 mg or rosuvastatin 40 mg is used in <5 % of dyslipidaemic patients [20–22]. Finally the question is whether we should expect that one drug (a statin) would completely reduce the overall risk? Probably the best answer to this question regarding the role of statins in secondary prevention is the fact that all available (ezetimibe, fenofibrate) and currently investigated (e.g. PCSK9 and CETP inhibitors) drugs are mainly considered for use ‘on top of’ statins.

The role of statins in primary prevention seems to also be well established [23]. The CTT meta-analysis of 14 randomized trials (90,056 individuals) showed that statins can safely reduce the 5-year incidence of major coronary events, coronary revascularization and stroke by about 12 % per 1 mmol/l reduction in LDL-C, irrespective of the initial lipid profile or other presenting characteristics [24]. The observed reduction in all-cause mortality (risk ratio (RR) 0.88, *P* <0.0001) reflected a 19 % reduction in coronary mortality (0.81, *P* <0.0001), with significant corresponding reductions in myocardial infarction or coronary death (0.77, *P* <0.0001), in the need for coronary revascularization (0.76, *P* <0.0001), in fatal or non-fatal stroke (0.83, *P* <0.0001), and, combining these, of 21 % in any such major vascular event (0.79, *P* <0.0001) [24]. Taking all years together, the overall reduction of about one fifth per mmol/l LDL-C reduction translated into 48 fewer participants having major vascular events per 1,000 among those with pre-existing coronary heart disease (CHD) at baseline, compared with 25 per 1,000 among participants with no such history [24].

These results were confirmed in a meta-analysis that included ten trials (70,388 participants without established CVD), which showed that statins significantly reduced the risk of all-cause mortality (odds ratio (OR) 0.88), major coronary events (0.70) and major cerebrovascular events (0.81) [25]. In another analysis, Ray et al. determined whether statin therapy reduces all-cause mortality among intermediate to high-risk individuals without a history of CVD [26]. Data from 11 studies with 65,229 participants showed that use of statins in the high-risk primary prevention setting was not associated with a statistically significant reduction (RR 0.91) in the risk of all-cause mortality. These findings complement and extend the above-mentioned meta-analyses [24] as well as the results from the Justification for the Use of Statins in Primary Prevention: An Intervention Trial Evaluating Rosuvastatin (JUPITER) trial [27], which reported that statins reduced death from any cause by 12 %. However, these estimates were based on information from both individuals with and without pre-existing CHD or stroke, which may overestimate the real benefits in the primary prevention setting. Despite the continuing discussion of the effect of statins on all-cause mortality in these groups of patients, their role in primary prevention individuals, especially in high-risk ones, is unquestionable. In low and moderate-risk patients the decision regarding starting statin therapy should be assessed individually taking into account the cardiovascular risk as well as the risk of statin-related side effects (e.g. new onset diabetes, especially in patients with overweight/obesity and/or insulin resistance) [28].

Statins have been called the drugs of 21st century [6]; this is connected both with their potent lipid-lowering properties as well as pleiotropic actions (e.g. anti-inflammatory, antioxidant and antithrombotic). These off-lipid-lowering properties have been the topic of considerable discussion; possibly these properties are simply the result of intense LDL-C reduction and as a consequence decrease inflammation and oxidative stress, and stabilize atheromatous plaques [29–31]. That is why within LBPMC Group we carried out a series of meta-analyses, which aimed to answer the question regarding the pleiotropic effects of statins. Endothelial dysfunction may be associated with increased circulating asymmetric dimethylarginine (ADMA) levels, an L-arginine analogue, which inhibits nitric oxide (NO) synthesis [29]. Therefore, we assessed the effect of statins on circulating ADMA levels [29]. In the analysis of nine randomized controlled trials (RCTs) with 1,134 participants we showed a significant reduction in plasma ADMA concentrations following statin therapy compared with placebo (−0.104 μM, *P* <0.0001) [29]. We also investigated the effect of statin therapy on circulating endothelin-1 (ET-1) levels [30]. Data from 15 RCTs showed that statin therapy significantly reduced plasma ET-1 concentrations (−0.30 pg/ml, *P* <0.01), and this effect was not affected by the duration of statin therapy (<12 weeks: −0.51 pg/ml, *P* <0.01; >12 weeks: −0.19 pg/ml, *P* <0.05) or by dose of statins (<40 mg/day: −0.27 pg/ml, *P* = 0.01; >40 mg/day: −0.38 pg/ml, *P* = 0.01) [30]. We also investigated the antithrombotic effects of statins, analysing the effect of statin therapy on D-dimer levels [31] and von Willebrand factor Antigen (vWF:Ag) [32]. In the first analysis of nine RCTs with 1,165 participants we showed a significant effect of statin therapy in reducing plasma D-dimer levels (−0.988 μg/ml, *P* = 0.001), with the effect significant only in the subsets of studies with treatment duration ≥12 weeks (−0.761 μg/ml, *P* <0.001) and for lipophilic statins (atorvastatin and simvastatin) (−1.364 μg/ml, *P* = 0.001) [31]. In the other meta-analysis we assessed the impact of statins on vWF:Ag levels [32]. vWF is involved in platelet adhesion, coagulation factor VIII binding and transport, and formation and deposition of thrombus, and elevated vWF concentrations have been shown to be associated with endothelial dysfunction and/or injury [32]. In the meta-analysis of 21 treatment arms we showed a significant decrease in plasma vWF:Ag levels following statin therapy (SMD −0.54, *P* = 0.001). In subgroup analyses, the effect size calculated for lipophilic statins was greater (−0.56, *P* = 0.003) than that of hydrophilic ones (−0.38, *P* = 0.046), and in the subset of studies lasting ≥12 weeks (−0.70, *P* = 0.005) compared with that of studies lasting <12 weeks (−0.34, *P* = 0.052) [32].

All these meta-analyses support the pleiotropic properties of statins and confirm the multifactorial mechanism of action of statins. These effects may lead to statins becoming the most important cardiovascular drugs, both in primary and secondary prevention settings.

## Is statin-modified reduction in lipids the most important preventive therapy for cardiovascular disease? No!

**Fig. 4:**
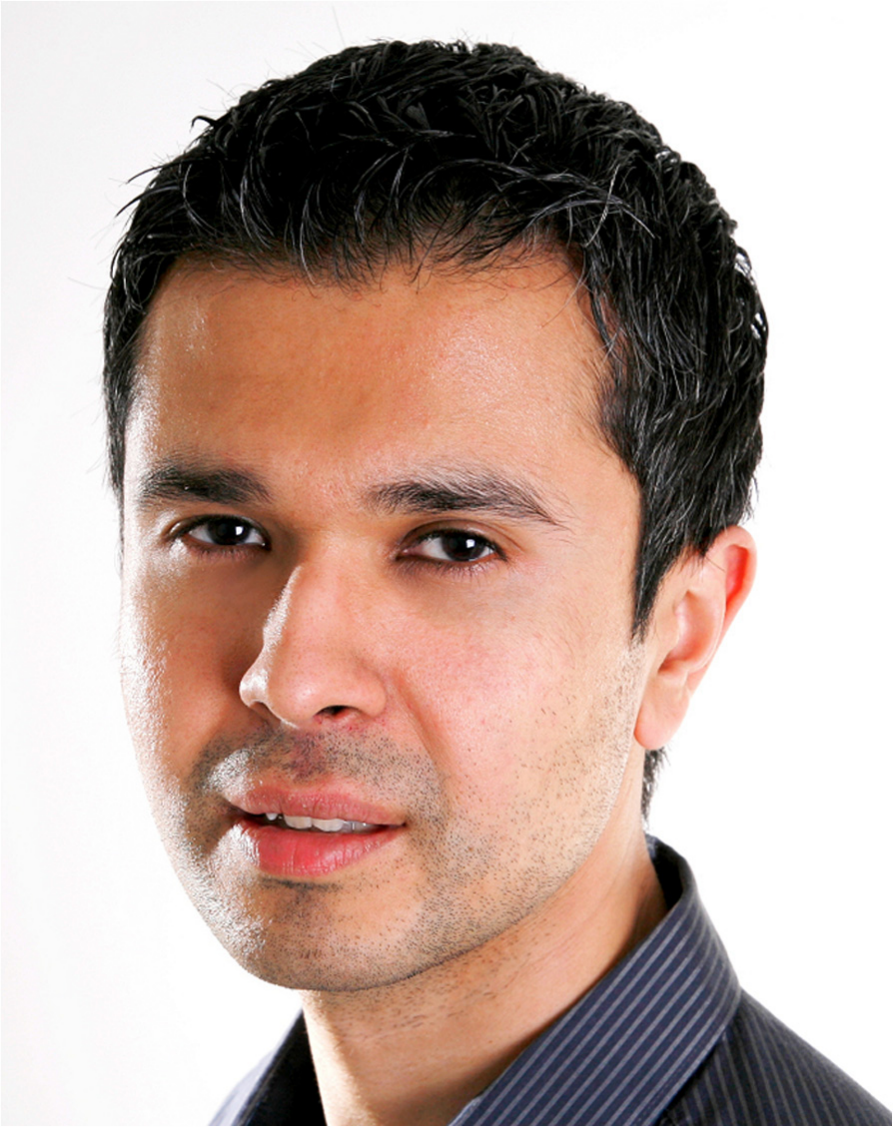
Aseem Malhotra is an Honorary Consultant Cardiologist at Frimley Park Hospital, Surrey, UK, and founding member and cardiologist advisor to the campaign group, Action on Sugar. He also serves on the external advisory board of Saving Londoners’ Lives (SLL). Dr Malhotra is widely regarded as a central figure in the debate on harms of excess sugar consumption. His research interests include preventative and personalized medicine, CVD, hypercholesterolaemia and hypertension

**Fig. 5:**
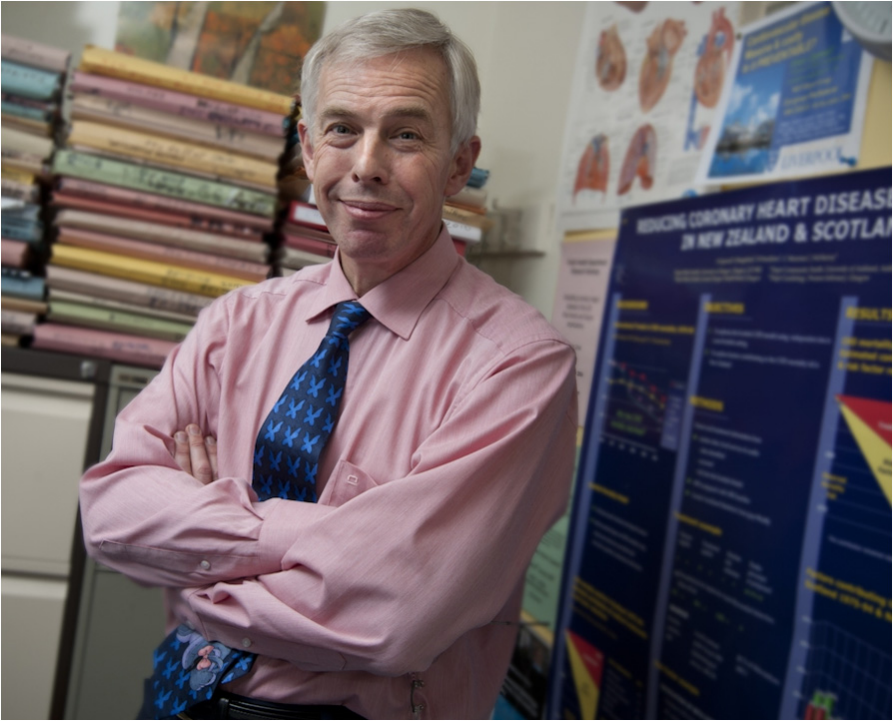
Simon Capewell is Professor of Clinical Epidemiology at the Department of Public Health and Policy, University of Liverpool, Liverpool, UK. He is President of the Society for Social Medicine and Vice President (Policy) of the Faculty of Public Health. His research interests include examining global trends in CVD prevalence, and developing effective and cost-saving CVD prevention strategies which build on empirical evidence, policy analyses and quantitative modelling in high, middle and low-income countries

Aseem Malhotra and Simon Capewell (Figs. 4 and 5)

The published literature states an unequivocal mortality benefit for secondary prevention – patients with established heart disease (absolute risk reduction (ARR) 1.2 %, NNT 83) over 5 years [33]. However, the same does not apply in primary prevention, especially in individuals at low risk. Independent analysis of the largest and most comprehensive meta-analysis carried out by the CTT Collaboration revealed no mortality benefit in individuals with a <20 % risk of developing CVD in the next 10 years [34]. With no significant mortality benefit the question then moves to non-mortality benefits, and potential harms. The same large analysis suggested a 1 in 140 risk reduction in non-fatal heart attack or stroke in those with <10 % risk of CVD over 10 years but no reduction in serious illness. For truly informed decision making in the prescription of statins, we believe it is an ethical imperative to estimate both the number needed to treat (NNT) and also the number needed to harm (NNH) [35, 36].

There are clearly understandable problems when relying on industry-sponsored trials designed primarily to determine the benefits of statins, to ascertain the true incidence of side effects that may interfere with the quality of life. This partly reflects the high selectivity of participants in clinical trials. Pre-randomization run-in periods may also be used to exclude patients who fail to tolerate statins. For example, 36 % of screened patients were excluded from the Heart Protection Study before the actual trial even began, which had the potential to screen out many patients who may have suffered from adverse effects from simvastatin, including muscle symptoms [37]. In community-based studies, over half of statin users will cease to take the drug within a year, with 62 % of those patients citing side effects as the reason [38, 39]. The uncontrolled nature of these large cohort studies is clearly not ideal. However, a recent article suggesting that in industry-sponsored RCTs there are no significant differences in the rates of adverse effects between active statins versus placebo groups is unhelpful [3]. This simply adds ‘false precision to biased estimates’ [40]. These trials have not adequately assessed the most commonly reported side effects reported by patients, notably muscle pains and cognitive impairment. The head of the CTT Collaboration has recently conceded that they did not assess for all possible side effects [41]. As every practising clinician knows, such side effects are common when patients are actually asked.

A publicly funded double-blinded RCT published in the *Archives of Internal Medicine* involving 1,016 low-risk patients receiving simvastatin 20 mg, pravastatin 40 mg or placebo revealed that both drugs had a significant adverse effect on energy/fatigue exercise score, with up to 40 % of women reporting reduced energy or fatigue with exertion [42].

Furthermore, it is instructive to note that Pfizer’s own patient information leaflet of atorvastatin states that ‘common side effects (may affect up to 1 in 10 patients)’ of the drug include pain in the throat, nausea, indigestion, joint and muscle pain, and increases in blood sugar levels [43]. Fortunately the majority of these symptoms may be reversible on cessation of the drug. However, the small increased 0.5–1.1 % risk of type 2 diabetes now directly attributed to statins should not be dismissed lightly.

Over 80 % of CVD is attributable to environmental factors, notably unhealthy diet and also smoking, alcohol and physical inactivity. Diet has primacy, accounting for a larger burden of CVD disease and death than tobacco, alcohol and inactivity combined [44].

For those at low risk eating an apple a day has an equivalent risk reduction for myocardial infarction as taking a statin [45]. In a recent large primary prevention RCT for those at high risk, a Mediterranean diet supplemented with 30 g of nuts daily or at least 4 tablespoons of extra virgin olive oil reduced the risk of cardiovascular events by almost a third [46]. There was an absolute risk reduction of 1.5 % (NNT 61) for stroke in comparison to Mediterranean diet alone and no adverse dietary effects noted. It is likely that these benefits might have been even larger if compared to the average Western diet.

Crucially, these dietary benefits are independent of differences in LDL-C. It is the abundant alpha-linoleic acid, polyphenols and omega-3 fatty acids provided in nuts, olive oil, oily fish, vegetables and fruit that rapidly exert positive health effects by attenuating inflammation, atherosclerosis and thrombosis [47]. Concentrating on LDL-lowering alone as an end in itself might therefore be counterproductive. Indeed, insulin resistance may emerge as the single most important determinant of coronary artery disease. In young adults, preventing insulin resistance could prevent 42 % of myocardial infarctions, a larger reduction than correcting hypertension (36 %), low high-density lipoprotein cholesterol (HDL-C) (31 %), body mass index (BMI) (21 %) or LDL-C (16 %) [48]. It is plausible that the small benefits of statins in the prevention of CVD come from pleiotropic effects which are independent of LDL-lowering. The focus in primary prevention should therefore be on foods and food groups that have a proven benefit in reducing hard endpoints and mortality.
